# Multitask bidirectional digital coding metasurface for independent controls of multiband and full-space electromagnetic waves

**DOI:** 10.1515/nanoph-2022-0190

**Published:** 2022-05-06

**Authors:** Liang Wei Wu, Hui Feng Ma, Yue Gou, Rui Yuan Wu, Zheng Xing Wang, Qiang Xiao, Tie Jun Cui

**Affiliations:** State Key Laboratory of Millimeter Waves, School of Information Science and Engineering, Southeast University, Nanjing, 210096, China; Institute of Electromagnetic Space, Southeast University, Nanjing, 210096, China

**Keywords:** bidirectional, digital coding metasurface, full space, multi-band, multi-task

## Abstract

Full-space metasurfaces demonstrate powerful abilities in manipulating electromagnetic (EM) waves, but most of them are usually single band. Here, a multiband bidirectional digital coding metasurface is proposed for multiple tasks, which can achieve completely different functions in up to six frequency bands when the EM waves are incident from the front and back of the metasurface. As a proof-of-concept, we design and fabricate a dual-band full-space metasurface with integrated functions of reflection, transmission, holographic imaging, and vortex-beam forming, and a six-band full-space metasurface with completely independent holographic imaging functions at different frequency bands. Simulated and measured results are in good agreements with the theoretical predictions, verifying the good performance of the proposed multitask bidirectional digital coding metasurface.

## 1 Introduction

Metasurface, as a two-dimensional (2D) planar version of metamaterial, provides an easy way to control the electromagnetic (EM) waves by properly engineering and arranging periodic or aperiodic subwavelength artificial meta-atoms [1], [2], [3], which can not only inherit the superior capabilities of metamaterials in manipulating the EM properties, such as phase [4], [5], [6], [7], magnitude [7], [8], [9], polarization [10, 11], but also overcome the challenges encountered in metamaterials, such as high losses, bulk, difficult manufacturing, and so on.

Generally, the metasurfaces are mainly classified to two types: reflection [4, 11], [12], [13] and transmission [6, 14], which have demonstrated powerful abilities to control the EM waves. However, most of them can only manipulate the EM waves in half space, including polarization-dependent metasurfaces [15], [16], [17], [18], which restrict the utilization of space resources. In order to expand the utilization of space resources and improve the information capacity, the concept of full-space metasurface has been proposed in recent years [19], [20], [21], [22], but they usually work in a single frequency or are direction dependent, which severely limit the design freedom. A spin-encoded wavelength-direction multitasking Janus metasurface was proposed in Ref. [23], but it can only achieve one single wavelength control of transmission waves whether the EM waves are incident from the front or the back [23]. In 2014, the concept of digital coding metasurface was proposed [24], [25], [26], which provides a link between the physical world and information science. Many impressive works have been proposed using digital coding metasurfaces, such as EM diffusion [27], microwave holography [28, 29], harmonic modulations [30], [31], [32], and wireless communications [33, 34]. The digital coding method simplifies the design and provides a new inspiration for the full-space metasurface [29, 35], [36], [37], [38], [39]. However, the previously proposed full-space digital coding metasurfaces still have similar limitations of single band and unidirectional control.

Here, we propose a multitask bidirectional digital coding metasurface that can achieve independent controls of both reflection and transmission waves in different frequency bands, and perform different functions when the EM waves are incident from the front and the back of the metasurface, respectively. By adjusting the geometric parameters of unit cells, the working frequency band can be customized and extended to six frequency bands at most, and the metasurface can perform completely different functions in each band. As a proof of concept, a dual-band full-space metasurface with integrated functions of reflectarray, transmitarray, holographic imaging and vortex beam generation, as well as a six-band full-space metasurface with independent holographic imaging function of Arabic numerals “1” to “6” at each frequency band are designed and fabricated. The measured results show a good agreement with the theoretical predictions and full-wave simulations, verifying the excellent performance of the proposed multitask bidirectional digital coding metasurface. This work may provide an efficient way to expand the utilization of space resources and improve information capacity of metadevices.

## 2 Principe of operation

**Figure 1:**
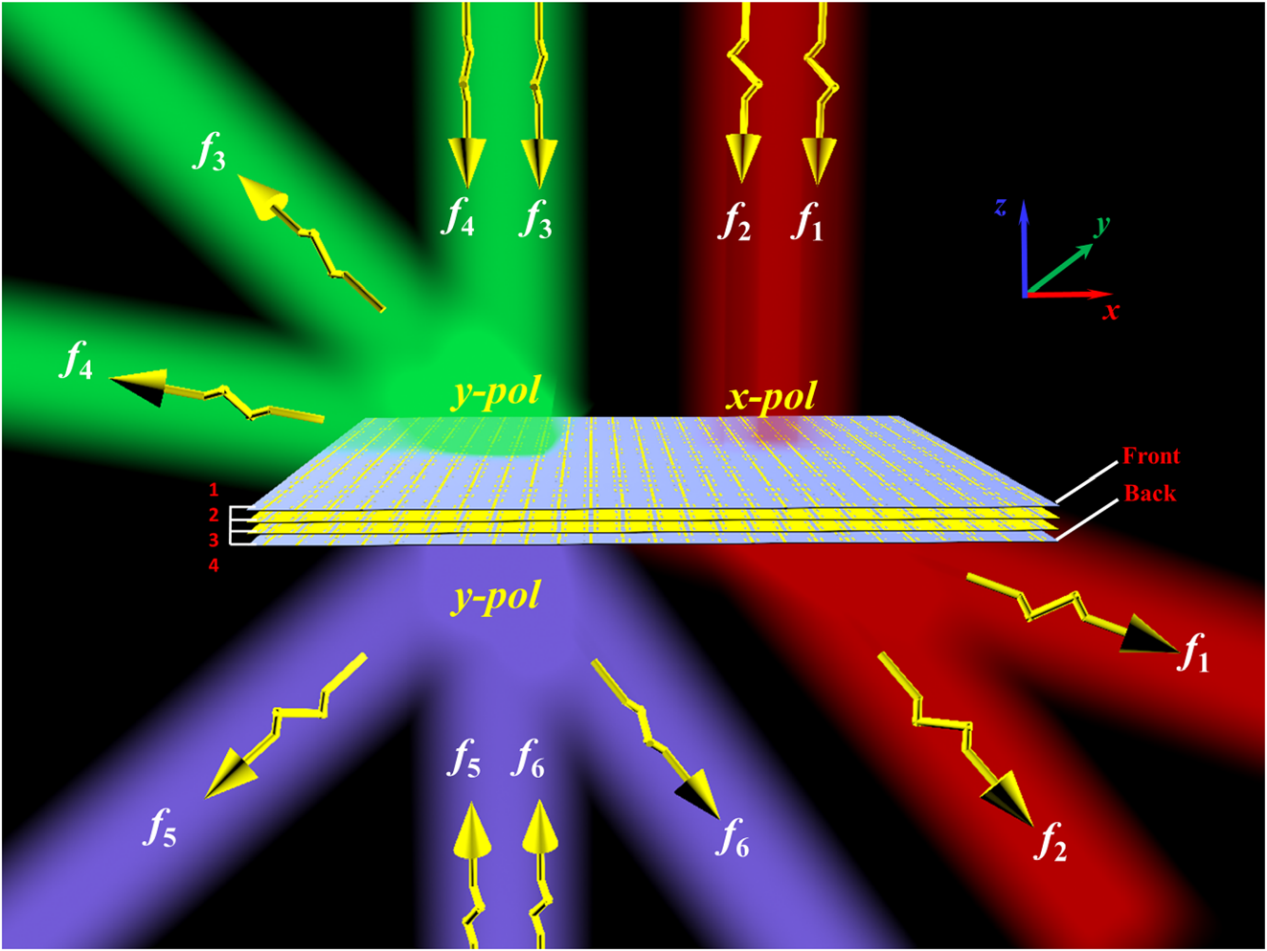
Schematic diagram of the proposed multitask bidirectional digital coding metasurface for independent control of multiband full-space EM waves.

Figure 1 shows the schematic of the proposed multi-task bidirectional full-space digital coding metasurface, which is composed of four layers. When the *x*-polarized EM waves are incident from the front of the metasurface (along −*z* direction), the EM waves can be effectively transmitted and independently controlled at frequencies of *f*_1_ and *f*_2_, respectively. Because of the structural reciprocity, the same conclusion can also be obtained when the *x*-polarized EM waves are incident from the back of the metasurface (along *z* direction), which is not shown in Figure 1. When *y*-polarized EM waves are incident from the front of the metasurface, the EM waves will be totally reflected at the layer 2 and independently controlled at frequencies of *f*_3_ and *f*_4_, respectively. Similarly, when *y*-polarized EM waves are incident from the back of the metasurface, the independent control of dual-band reflection waves at other two different frequencies of *f*_5_ and *f*_6_ can be achieved. In addition, by adjusting the corresponding geometric parameters of unit cells, these three groups of working frequencies ((*f*_1_, *f*_2_), (*f*_3_, *f*_4_), (*f*_5_, *f*_6_)) can be designed to be the same or totally different. As a proof of concept, a dual-band full-space metasurface with multifunctional integration of reflectarray, transmitarray, holographic imaging and vortex beam generation, and a six-band full-space metasurface with independent holographic imaging function at six different frequencies are designed and fabricated, respectively.

**Figure 2:**
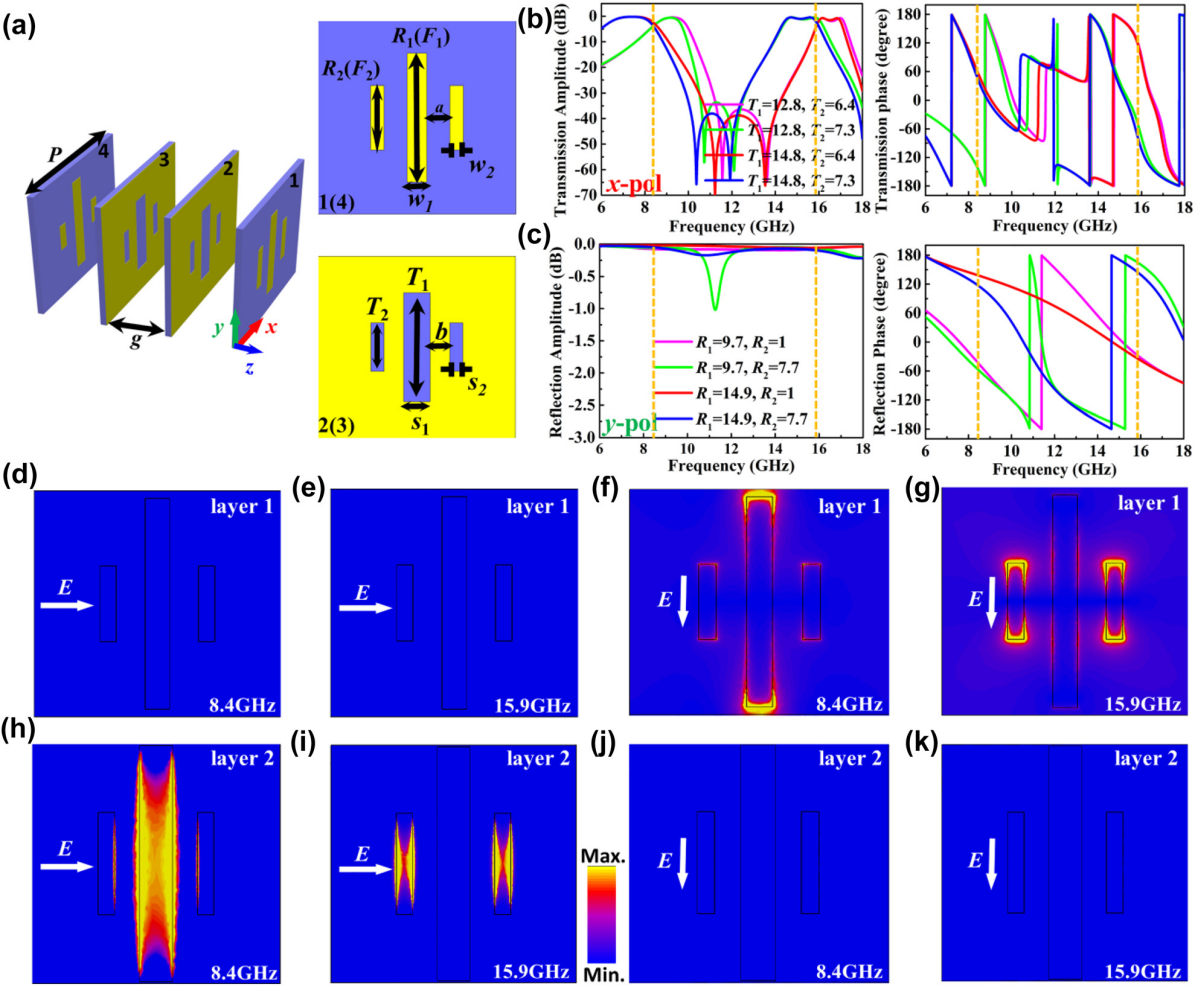
The configuration of unit cell and its EM responses. (a) The configuration of unit cell. (b, c) The amplitude and phase characteristics of the unit cell under (b) *x*- and (c) *y*-polarized incidences. (d–g) The simulated amplitude distribution of electric fields on the first layer with *R*_1_ = 14 mm, *R*_2_ = 5 mm under *x*-polarized incidence at (d) 8.4 GHz and (e) 15.9 GHz, and under *y*-polarized incidence at (f) 8.4 GHz and (g) 15.9 GHz. (h–k) The simulated amplitude distribution of electric field on the second layer with *T*_1_ = 14.8 mm, *T*_2_ = 6.4 mm under *x*-polarized incidence at (h) 8.4 GHz and (i) 15.9 GHz, and under *y*-polarized incidence at (j) 8.4 GHz and (k) 15.9 GHz.

The unit cell of metasurface is illustrated in Figure 2a, which is a four layer structure. The layer 1 and 4 are similar and composed of three metal strips, while the layer 2 and layer 3 are the same with three slits etched on a metal surface. The dielectric substrates are Rogers 4350B with relative permittivity of 3.66 and loss tangent of 0.0037, whose thickness is 0.508 mm. The air spacing between the adjacent dielectric substrates is *g* = 5 mm, and the period of unit cell is p = 15 mm. The other geometric parameters of *a*, *b*, *w*_1_, *w*_2_, *s*_1_, *s*_2_, *R*_1_, *R*_2_, *F*_1_, *F*_2_, *T*_1,_ and *T*_2_ are variables, which can be designed according to the working frequencies and functionalities of metasurface. It is worth mentioning that the parameters of *R* (*R*_1_, *R*_2_) and *F* (*F*_1_, *F*_2_) represent the lengths of metal strips on the first and fourth layers, respectively.

For the first case, to design a dual-band full-space metasurface, we first fix partial parameters as follows: *a* = 1.75 mm, *b* = 1.5 mm, *w*_1_ = 1.5 mm, *w*_2_ = 1 mm, *s*_1_ = 2 mm, and *s*_2_ = 1 mm, and then adjust the *R*_1_, *R*_2_, *F*_1_, *F*_2_, *T*_1,_ and *T*_2_ to meet the required transmission and reflection phases. Here, the first and fourth layers are set to be the same, i. e. *R*_1_ = *F*_1_ and *R*_2_ = *F*_2_. Figure 2b shows the transmission amplitude and phase distributions of unit cell under *x*-polarized incidence with the changes of *T*_1_ and *T*_2_. The unit cell has the high transmittance at both 8.4 GHz (−3.2 dB) and 15.9 GHz (−2.8 dB), while the 180° transmission phase differences at 8.4 and 15.9 GHz can be achieved by changing *T*_1_ and *T*_2_, respectively. Hence, according to the knowledge of coding metamaterials [24], the unit cell can be used to realize dual-band 1-bit transmission phase code to independently manipulate the transmitted *x*-polarized waves at 8.4 and 15.9 GHz, respectively. However, when *y*-polarized waves are incident along −*z* direction, the waves will be totally reflected by unit cell, and the 180° reflection phase differences at 8.4 and 15.9 GHz also can be achieved by changing *R*_1_ and *R*_2_, respectively, as shown in Figure 2c. Hence, dual-band 1-bit reflection phase code can be realized to independently manipulate the reflected *y*-polarized waves at 8.4 and 15.9 GHz, respectively. It should be pointed out that the efficiency of transmission mode is only about 50% due to the limitation of transmission amplitude, but the efficiency of reflection mode can be as high as 97% because the reflection amplitude can reach near to 100%.

To illustrate the working mechanism of unit cell, the simulated amplitude responses of electric fields on the layer 1 and 2 are given in Figure 2d–k for *x*- and *y*-polarized waves incident from the front of unit cell, respectively. For *x*-polarized waves, metal strips on layer 1 almost have no EM responses at both 8.4 and 15.9 GHz, as shown in Figure 2d and e, while the strong resonances are excited at 8.4 and 15.9 GHz by the long and short slits on layer 2, respectively, as shown in Figure 2h and i. Hence, because layers 2 and 3 are the same, the amplitude and phase responses of *x*-polarized transmission waves at 8.4 and 15.9 GHz are mainly related to the long and short slits on these two layers, respectively, which are consistent with the results shown in Figure 2b. For *y*-polarized waves, the strong resonances are excited by the long and short metal strips on layer 1 at 8.4 and 15.9 GHz, respectively, as shown in Figure 2f and g, while no any EM response is excited by layer 2, as shown in Figure 2j and k. Considering that the layer 2 is composed of metal sheet decorated with slits, no EM response means all the incoming waves are totally reflected, so the phase responses of *y*-polarized reflection waves at 8.4 and 15.9 GHz are mainly dependent on the long and short metal strips on layer 1, respectively, which are consistent with the results shown in Figure 2c. The same conclusion can be made when *y*-polarized waves incoming from back side, and because the *y*-polarized waves incoming from front and back sides are isolated by layers 2 and 3, the forward and backward reflection waves can be independently manipulated.

**Figure 3:**
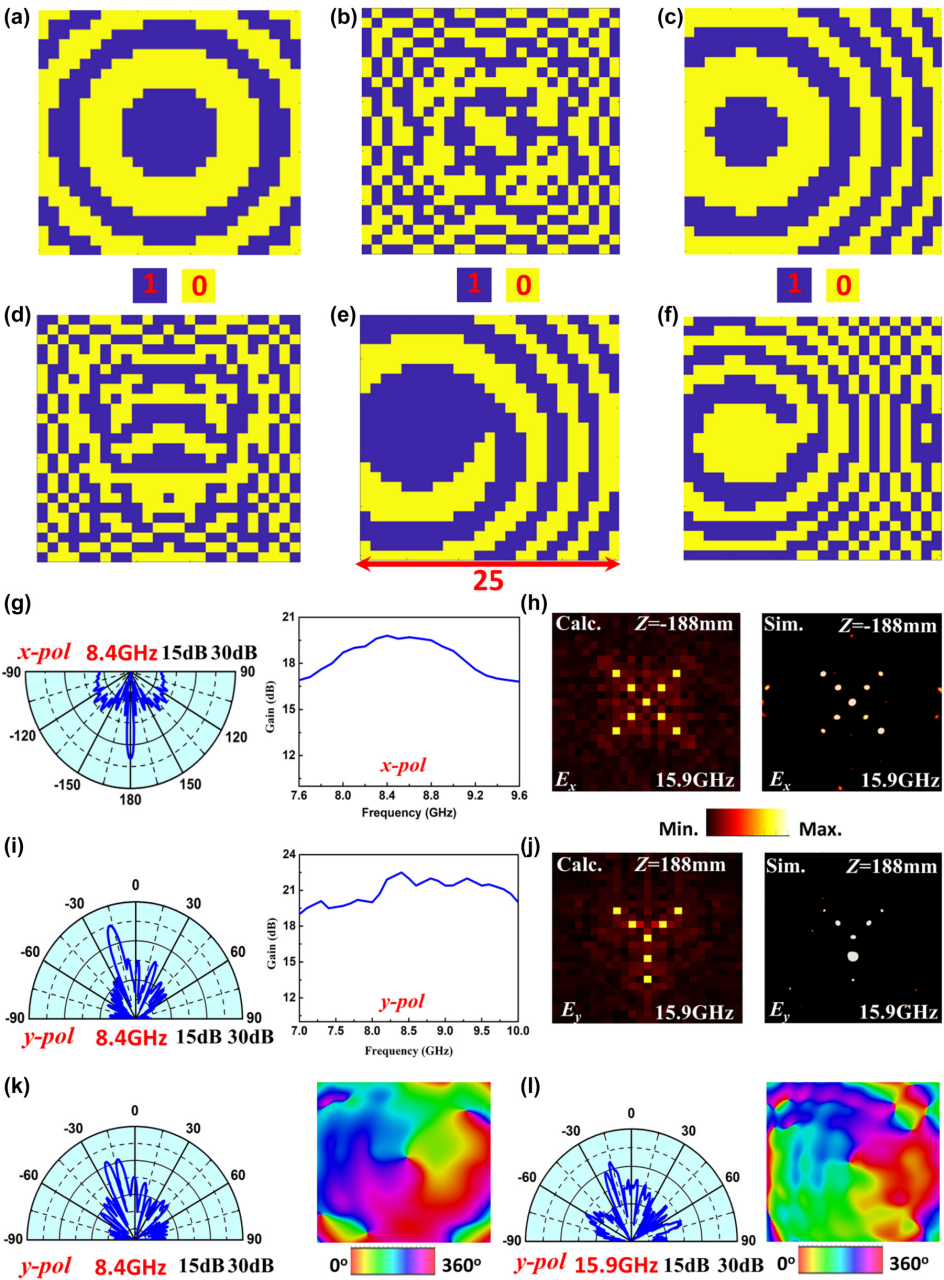
The phase distributions and simulated results of the dual-band full-space digital coding metasurface. (a–f) The 1-bit phase distribution of (a) the transmitarray at 8.4 GHz, (b) the transmitted imaging at 15.9 GHz, (c) the reflectarray at 8.4 GHz, (d) the reflected imaging at 15.9 GHz, (e) the reflected beam with *l* = +1 OAM at 8.4 GHz and (f) the reflected beam with *l* = −1 OAM at 15.9 GHz. (g) 2D far-field radiation pattern and gain bandwidth of the transmitarray under the *x*-polarized incidence. (h) The holographic images of letter “X” on the plane of *z* = −188mm at 15.9 GHz. (i) 2D far-field radiation pattern and gain bandwidth of the reflectarray under the *y*-polarized incidence. (j) The holographic images of letter “Y” on the plane of *z* = 188 mm at 15.9 GHz. (k–l) 2D far-field radiation patterns and phase distributions of (k) *l* = +1 OAM beam and (l) *l* = −1 OAM beam on the plane of *z* = −600mm under the *y*-polarized incidence along *z* direction.

Based on the above mentioned unit cell, a dual-band full-space metasurface with multi-functional integration of reflectarray, transmitarray, holographic imaging, and vortex beam generation is designed and simulated. The metasurface is composed of 25 × 25 unit cells and designed to work at 8.4 and 15.9 GHz, respectively. Figure 3 shows the distributions of coding sequences for different functions and the simulated results.

For the *x*-polarized waves, the transmitted directive radiation and holographic imaging are constructed at 8.4 and 15.9 GHz, respectively. For directive radiation at 8.4 GHz, an *x*-polarized rectangular waveguide antenna as a feeding source of transmitarray is placed 300 mm away from the center of metasurface, and the corresponding calculated 1-bit transmission phase distribution is given in Figure 3a. The simulated far-field radiation pattern and gain are demonstrated in Figure 3g. The result shows that the maximum gain of 19.8 dB and the side lobe level of −14.3 dB can be achieved at 8.4 GHz, whose 3-dB gain bandwidth is 2 GHz (23.8%). For holographic imaging at 15.9 GHz, an *x*-polarized plane waves are normally incident to the metasurface along −z direction, and the focal plane of transmission holographic image is designed at *z* = −188mm, and the 1-bit holographic phase distribution of a letter “X” is given in Figure 3b, which is calculated by the weighted Gerchberg–Saxton algorithm [40]. The simulated holographic image has a good agreement with the theoretical calculation, as shown in Figure 3h.

For the *y*-polarized waves, when they are incident from the front of metasurface, the reflected directive radiation and holographic imaging are constructed at 8.4 and 15.9 GHz, respectively. For directive radiation at 8.4 GHz, a *y*-polarized rectangular waveguide as a feeding source of reflectarray is placed 300 mm away from the center of metasurface, and the reflected beam is deflected to the direction of −17° in the *x*o*z* plane whose calculated 1-bit reflection phase distribution is given in Figure 3c. The simulated far-field radiation pattern and gain are shown in Figure 3i. The result verifies that the EM waves are reflected and deflected to −17°, with gain of 22.5 dB and side lobe level of −14.5 dB, whose 3-dB gain bandwidth can reach 2.9 GHz (34.5%). For holographic imaging at 15.9 GHz, the required 1-bit reflection phase distribution of a holographic image of letter “Y” under the normally incident plane waves is shown in Figure 3d, whose focal plane is *z* = 188 mm. The simulated result is also in good agreement with the calculations, as shown in Figure 3j. When the *y*-polarized EM waves are incident from the back side of metasurface, the reflected orbital-angular-momentum (OAM) beams with topological charges of *l* = +1 and *l* = −1 are realized at 8.4 and 15.9 GHz, respectively. The incident waves are generated by a rectangular wave guide placed 300 mm away from the center of metasurface, and the reflected beam is deflected to −17° in the *x*o*z* plane. Figure 3e and f show the 1-bit reflection phase distributions at 8.4 and 15.9 GHz, and the simulated far-field radiation patterns and near-field phase distributions of reflection beams are illustrated in Figure 3k and l, respectively, which verify that +1st-order and −1st-order OAM beams are generated at 8.4 and 15.9 GHz, respectively. Hence, six different functions are achieved by metasurface at two frequencies of 8.4 and 15.9 GHz.

**Figure 4:**
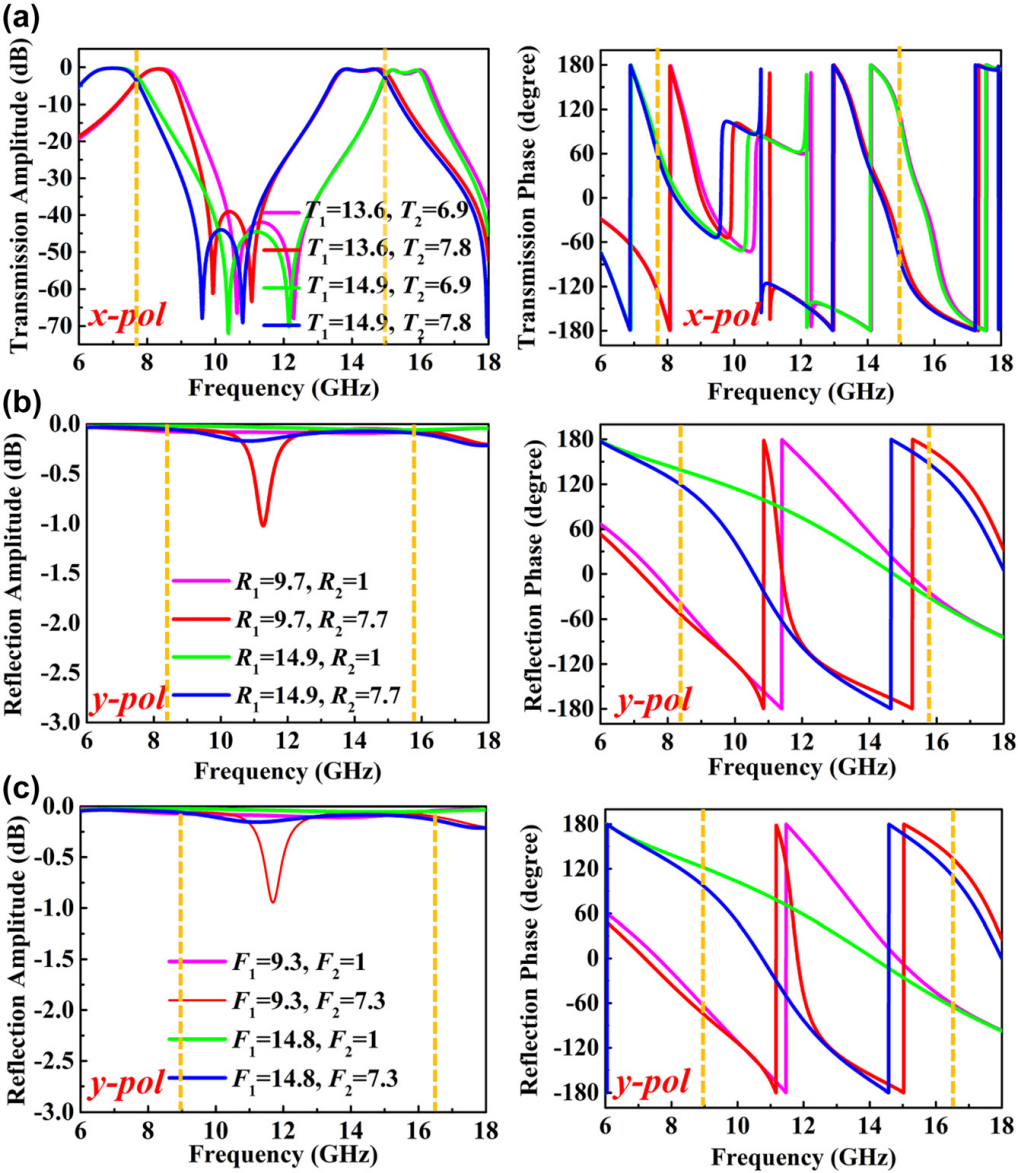
The unit cell characteristics of the six-band full-space digital coding metasurface. (a–c) The transmission amplitude and phase characteristics for (a) *x*-polarized incidences along −*z* direction, (b) *y*-polarized incidences along −*z* direction, and (c) *y*-polarized incidences along *z* direction.

According to the electric-field responses demonstrated in Figure 2d–k, three couples of working frequencies (*f*_1_, *f*_2_), (*f*_3_, *f*_4_) and (*f*_5_, *f*_6_) can be almost independently controlled by geometric parameters (*R*_1_, *R*_2_), (*F*_1_, *F*_2_), and (*T*_1_, *T*_2_), respectively. Hence, the metasurface can actually be designed to work at up to six completely different frequencies by adjusting these parameters. Based on this point, a metasurface that can work at six different frequencies has been further designed and demonstrated. Figure 4a–C) shows the amplitude and phase responses of unit cell to *x*-polarized transmission waves, *y*-polarized reflection waves along −z direction (front side) and *y*-polarized reflection waves along +z direction (back side), respectively, in which the partial geometric parameters of unit cell are fixed as *a* = 1.75 mm, *b* = 2.2 mm, *w*_1_ = 1.5 mm, *w*_2_ = 1 mm, *s*_1_ = 1 mm, and *s*_2_ = 0.6 mm. The results show that dual-band 1-bit transmission metasurface can be constructed at 7.7 and 15 GHz for *x*-polarized wave by changing the variables *T*_1_ and *T*_2_, as shown in Figure 4a, and dual-band 1-bit reflection metasurface can be constructed at 8.4 and 15.9 GHz for *y*-polarized waves incident from the front side by changing the variables *R*_1_ and *R*_2_, as shown in Figure 4b, and another dual-band 1-bit reflection metasurface can be constructed at 9 and 16.5 GHz for *y*-polarized waves incident from the back side by changing the variables *F*_1_ and *F*_2_, as shown in Figure 4c.

**Figure 5:**
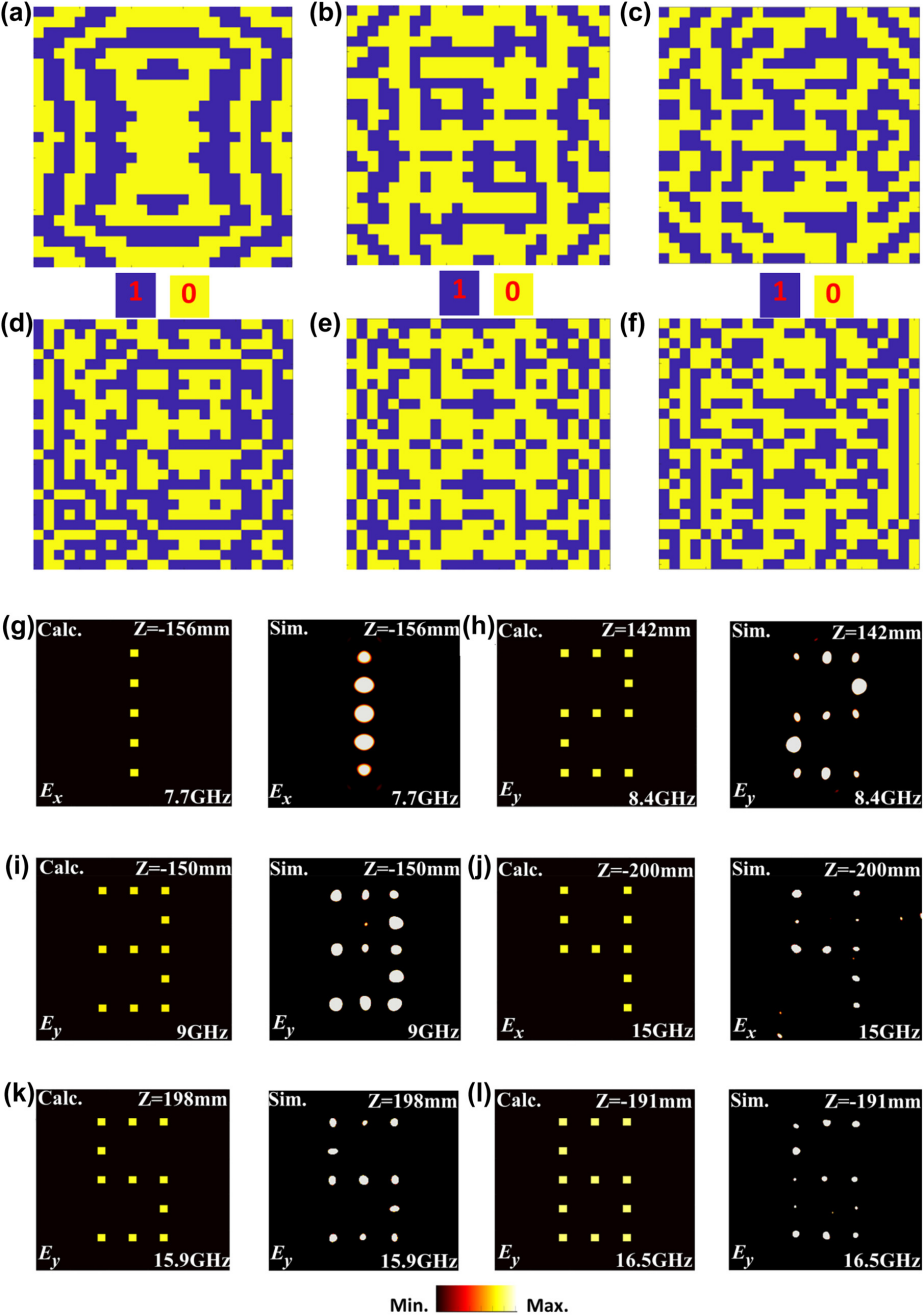
The phase distributions and holographic imaging results of the six-band full-space digital coding metasurface. (a–f) The 1-bit phase distributions for (a) transmission imaging of numeral “1” at 7.7 GHz, (b) reflection imaging of numeral “2” at 8.4 GHz, (c) reflection imaging of numeral “3” at 9 GHz, (d) transmission imaging of numeral “4” at 15 GHz, (e) reflection imaging of numeral “5” at 15.9 GHz, (f) reflection imaging of numeral “6” at 16.5 GHz. (g–l) The calculated and simulated results of holographic images of (g) numeral “1” at 7.7 GHz, (h) numeral “2” at 8.4 GHz, (i) numeral “3” at 9 GHz, (j) numeral “4” at 15 GHz, (k) numeral “5” at 15.9 GHz, and (l) numeral “6” at 16.5 GHz.

A six-band full-space metasurface composed of 25 × 25 unit cells has been designed to verify the proposed concept, which can achieve independent holographic images of Arabic numerals “1” to “6” at six different frequencies. Figure 5a–f show the required 1-bit phase distributions of metasurface for the holographic images of Arabic numeral “1”, “2”, “3”, “4”, “5”, and “6” at 7.7, 8.4, 9, 15, 15.9 and 16.5 GHz, respectively. The transmission holographic images of Arabic numerals “1” and “4” at 7.7 and 15 GHz for *x*-polarized waves are illustrated in Figure 5g and j, respectively, the reflection holographic images of Arabic numerals “2” and “5” at 8.4 and 15.9 GHz for *y*-polarized waves in forward space are illustrated in Figure 5h and k, respectively, and the reflection holographic images of Arabic numerals “3” and “6” at 8.4 and 15.9 GHz for *y*-polarized waves in backward space are illustrated in Figure 5i and l, respectively. All the simulated results show a good agreement with the calculations, verifying the ability of metasurface to independently work at six different frequencies. It is worth mentioning that all holographic images are designed in different focal planes, as marked in Figure 5g–l.

## 3 Experimental verification

**Figure 6:**
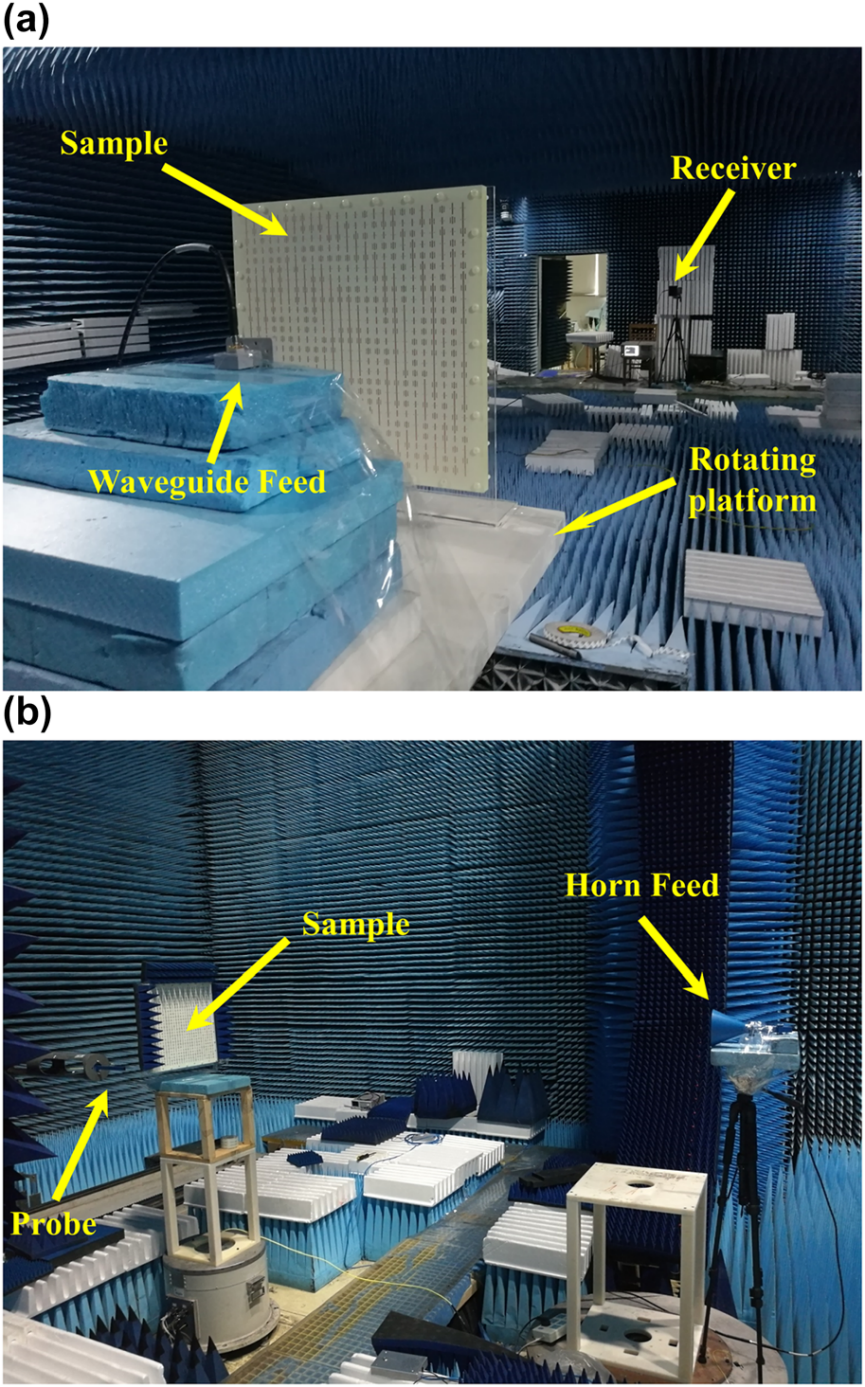
The experiment setups of the far-field and near-field experiments. (a) The far-field experiment setup. (b) The near-field experiment setup.

The above-mentioned two metasurfaces are fabricated and measured, respectively. Figure 6a and b illustrate the experimental setups of the far-field and near-field measurements, respectively. In the far-field experiment, the metasurface sample is placed on a rotational platform together with a rectangular waveguide feed. The distance between the rectangular waveguide feed and metasurface is 300 mm for both transmitarray and reflectarray. A receiving horn antenna (Receiver) is placed 15 m away from the metasurface to receive the far-field signal, as shown in Figure 6a. In the near-field experiment, a feeding horn is placed 3 m away from the sample to generate normally incident plane waves and a rectangular wave probe is located at focal planes of holographic images to measure near-field electric-field distributions, as shown in Figure 6b. The absorbing materials are wrapped around the sample to reduce the influence of edge diffraction introduced by incident waves.

**Figure 7:**
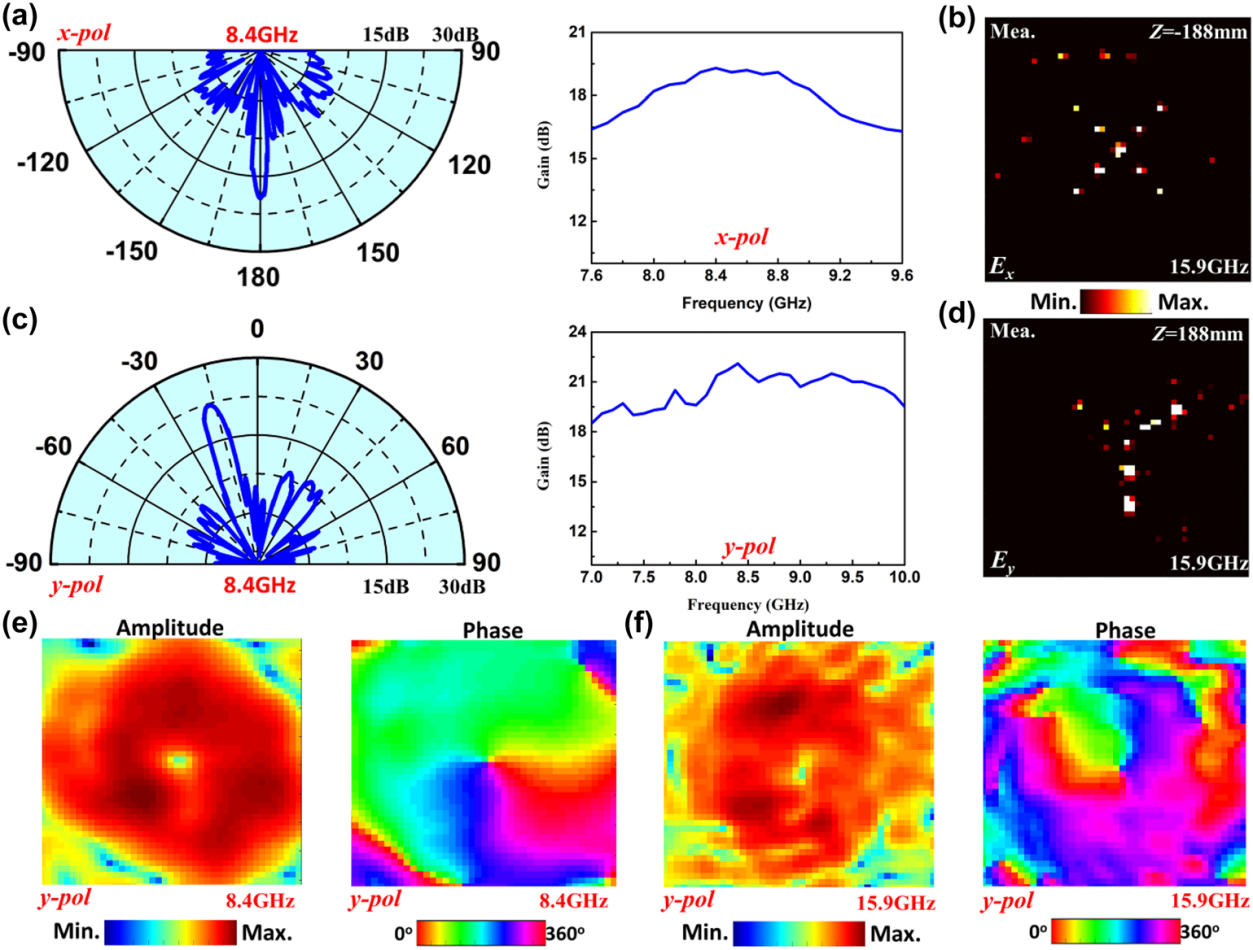
The experimental results of the dual-band full space digital coding metasurface. (a) 2D far-field and gain bandwidth of the 1 bit transmitarray under the *x*-polarized incidence. (b) The holographic image of letter “X” on the plane of *z* = −188mm at 15.9 GHz. (c) 2D far-field and gain bandwidth of the 1 bit reflectarray under the *y*-polarized incidence. (d) The holographic image of letter “Y” on the plane of *z* = 188 mm at 15.9 GHz. (e, f) The amplitude and phase distributions of (e) *l* = +1 OAM beam at 8.4 GHz, and (f) *l* = −1 OAM beam at 15.9 GHz.

Figure 7 demonstrates the experimental results of dual-band full-space digital coding metasurface. For *x*-polarized incidence along −z direction, the measured far-field radiation pattern of metasurface as a directive transmitarray at 8.4 GHz is illustrated in the left figure of Figure 7a, which shows that the radiation gain is 19.4 dB and side lobe is −12.9 dB. The 3-dB gain bandwidth can reach 2 GHz, as shown in the right figure of Figure 7a. The measured near-field distribution of transmission waves at 15.9 GHz is illustrated in Figure 7b. An expected holographic image of letter “X” is obtained at the focal plane of z = −188mm. For the *y*-polarized incidence along the −z direction, the measured far-field radiation pattern of metasurface as a directive reflectarray at 8.4 GHz is shown in the left figure of Figure 7c, showing that the radiation beam is deflected to *θ* = −17° with radiation gain of 22.1 dB and side lobe of −13.4 dB. The 3-dB gain bandwidth can reach 2.8 GHz as shown in the right figure of Figure 7c. The measured near-field distribution of reflection waves at 15.9 GHz is illustrated in Figure 7d. An expected holographic image of letter “Y” is obtained at the focal plane of *z* = 188 mm. For *y*-polarized incidence along +*z* direction, the OAM beams with topological charges of *l* = +1 and *l* = −1 are generated at 8.4 and 15.9 GHz, whose measured amplitude and phase distributions on the plane of *z* = −600 mm are shown in Figure 7e and f, respectively. All the far-field and near-field measured results are in good agreement with the calculated and simulated results shown in Figure 3.

**Figure 8:**
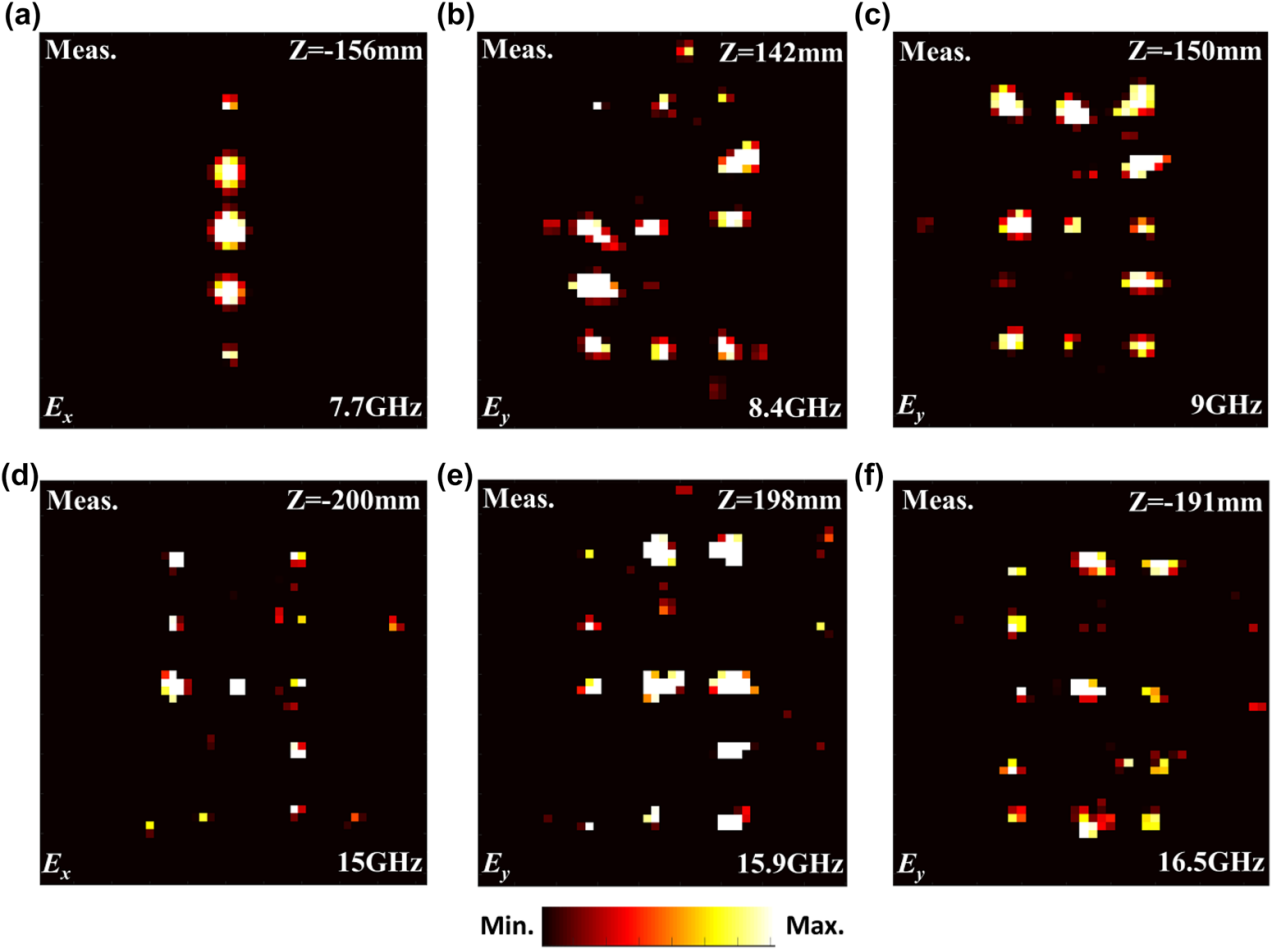
The experimental results of the six-band full space digital coding metasurface. (a) Numeral “1” at 7.7 GHz. (b) Numeral “2” at 8.4 GHz. (c) Numeral “3” at 9 GHz. (d) Numeral “4” at 15 GHz. (e) Numeral “5” at 15.9 GHz. (f) Numeral “6” at 16.5 GHz.

Figure 8 illustrates the experimental results of the six-band full-space metasurface. For the *x*-polarized incidence, the measured transmission Arabic numerals “1” and “4” on focal planes of *z* = −156mm and *z* = −200mm at 7.7 and 15 GHz are illustrated in Figure 8a and d, respectively. For *y*-polarized incidence along −*z* direction, the measured transmission Arabic numerals “2” and “5” on focal planes of *z* = 142 mm and *z* = 198 mm at 8.4 and 15.9 GHz are illustrated in Figure 8b and e, respectively. For *y*-polarized incidence along +*z* direction, the measured reflection Arabic numerals “3” and “6” on focal planes of *z* = −150 mm and *z* = −191 mm at 9 and 16.5 GHz are illustrated in Figure 8c and f, respectively. All the measured results are in good agreement with the calculated and simulated results shown in Figure 5, except for some slight differences, which may be caused by the accuracy of sample processing, assembly accuracy of multilayer plates and accuracy of quasi plane wave generated by feeding horn in experiment.

## 4 Conclusions

We have presented a multitask bidirectional digital coding metasurface that can achieve the independent multi-band controls of both reflection and transmission waves whether the EM waves are incident from the front or back of the metasurface. Further, the metasurface can independently work in up to six frequency bands in full space at the same time with completely different functions. As proofs of concept, a dual-band full-space metasurface and a six-band full-space metasurface are both simulated and fabricated with good performances. In addition, the coding of metasurface can also be easily extended to 2 bit by increasing the layers of unit structure. The proposed multitask bidirectional digital coding metasurface has powerful ability in manipulation of both reflection and transmission EM waves, which is incapable for previously reported metasurfaces and may provide a simple way to extend the functionality and information capacity of high-efficiency metadevices.
